# The Pathogenic TSH β-Subunit Variant C105Vfs114X Causes a Modified Signaling Profile at TSHR

**DOI:** 10.3390/ijms20225564

**Published:** 2019-11-07

**Authors:** Laura Kalveram, Gunnar Kleinau, Kamila Szymańska, Patrick Scheerer, Adolfo Rivero-Müller, Annette Grüters-Kieslich, Heike Biebermann

**Affiliations:** 1 Institute of Experimental Pediatric Endocrinology, Charité-Universitätsmedizin Berlin, corporate member of Freie Universität Berlin, Humboldt-Universität zu Berlin, and Berlin Institute of Health, 13353 Berlin, Germany; laura.kalveram@charite.de; 2 Institute of Medical Physics and Biophysics, Group Protein X-ray Crystallography and Signal Transduction, Charité – Universitätsmedizin Berlin, corporate member of Freie Universität Berlin, Humboldt-Universität zu Berlin, and Berlin Institute of Health, 10117 Berlin, Germany; gunnar.kleinau@charite.de (G.K.); patrick.scheerer@charite.de (P.S.); 3 Department of Biochemistry and Molecular Biology, Medical University of Lublin, 20-093 Lublin, Poland; kamila.szymanska@umlub.pl (K.S.); adolfo.rivero-muller@umlub.pl (A.R.-M.)

**Keywords:** central congenital hypothyroidism, G-protein coupled receptors, thyroid-stimulating hormone, TSHR

## Abstract

(1) Background: Central congenital hypothyroidism (CCH) is a rare endocrine disorder that can be caused by mutations in the β-subunit of thyrotropin (*TSHB*). The *TSHB* mutation C105Vfs114X leads to isolated thyroid-stimulating-hormone-(TSH)-deficiency and results in a severe phenotype. The aim of this study was to gain more insight into the underlying molecular mechanism and the functional effects of this mutation based on two assumptions: a) the three-dimensional (3D) structure of TSH should be modified with the C105V substitution, and/or b) whether the C-terminal modifications lead to signaling differences. (2) Methods: wild-type (WT) and different mutants of hTSH were generated in human embryonic kidney 293 cells (HEK293 cells) and TSH preparations were used to stimulate thyrotropin receptor (TSHR) stably transfected into follicular thyroid cancer cells (FTC133-TSHR cells) and transiently transfected into HEK293 cells. Functional characterization was performed by determination of Gs, mitogen activated protein kinase (MAPK) and Gq/11 activation. (3) Results: The patient mutation C105Vfs114X and further designed TSH mutants diminished cyclic adenosine monophosphate (cAMP) signaling activity. Surprisingly, MAPK signaling for all mutants was comparable to WT, while none of the mutants induced PLC activation. (4) Conclusion: We characterized the patient mutation C105Vfs114X concerning different signaling pathways. We identified a strong decrease of cAMP signaling induction and speculate that this could, in combination with diverse signaling regarding the other pathways, accounting for the patient’s severe phenotype.

## 1. Introduction

**Figure 1:**
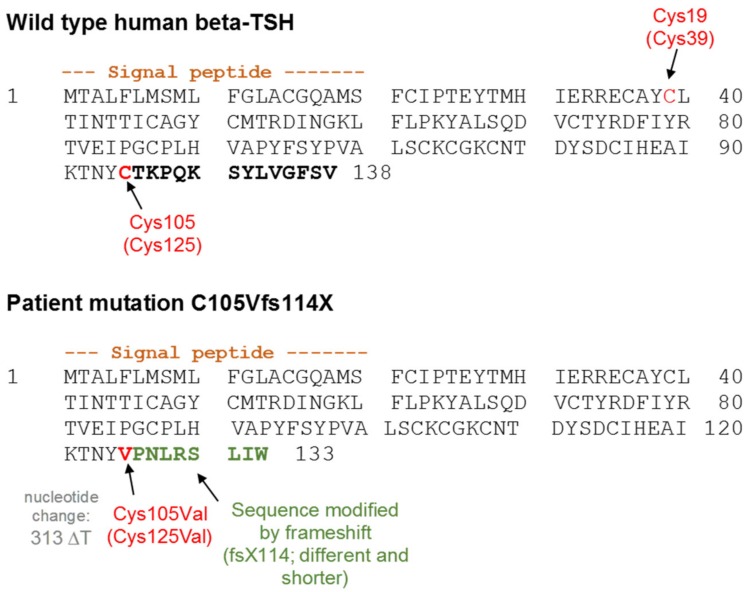
Comparison of the sequence of WT human TSHB and the patient mutation C105Vfs114X. Cysteine 19 and the cysteine at position 105 usually form a disulfide-bridge in WT TSH and are shown in red. The T-deletion and the resulting frameshift of the patient mutation leads to a replacement of the cysteine at position 105 by a valine (also shown in red). In addition, the following amino acids of the mutation (shown in green) differ from the WT (shown in bold black) and a premature stop results at position 114. Numbering with signal peptide is shown in brackets.

Central congenital hypothyroidism (CCH) is a rare but severe endocrine disorder with an incidence of about 1:50,000 [1]. One of the molecular causes for CCH are mutations in the *TSHB*-gene, which cause isolated TSH deficiency [2]. Of note, patients with the naturally occurring mutation c.313delT (C105Vfs114X) in the *TSHB*-gene show extraordinary early and severe signs of congenital hypothyroidism [3]. This is surprising, because it has previously been assumed that mutations in the *TSHB*-gene do not result in a severe phenotype, since the complete absence of TSH in patients with mutations in the *PIT-1* gene or *PROP-1* gene does not demonstrate a severe phenotype in most cases [4,5,6,7]. This has been attributed to the basal signaling activity (cAMP) of the TSHR, which may compensate for the absence of TSH induced signaling [8,9,10]. The mutation C105Vfs114X was first described in 1996, and so far 36 cases have been reported [11,12,13,14,15,16,17,18,19,20,21,22,23,24,25]. This mutation is caused by a T-deletion at nucleotide position 313 [11], resulting in a frameshift with a substitution of cysteine by valine at position 105 (amino acid sequence numbering without signal peptide) followed by eight non-homologous amino acids and a premature stop codon, thus lacking the five terminal WT amino acids (Figure 1) [26]. In other words, the 14 C-terminal (Ctt) amino acids in the WT are being exchanged by nine non-homologous amino acids (including the cysteine at position 105) in the C105Vfs114X mutation.

Within the first weeks to months of life, affected patients present with severe signs of congenital hypothyroidism, including hypothermia, lethargy, prolonged jaundice, muscle hypotonia, constipation and umbilical hernias. Furthermore, the hypothyroid state leads to delayed closure of the fontanelles as well as a delayed bone maturation. If the diagnosis and treatment with L-thyroxine is delayed, patients suffer from long-term psychomotor and neurocognitive deficiencies [13,15,16,19,21,25]. Therefore, patients with a *TSHB*-gene mutation show a severe phenotype comparable to patients with athyreosis [3,27]. The degree of pathology seen in *TSHB* mutations is incompletely understood, but may be attributed to (a) modifications in the TSHB protein structure and assembling of the glycoprotein hormone subunits (CGA- and TSHB-subunits), and/or (b) changes in signaling capacity at the TSHR (reviewed and described in detail in [26]). 

In brief, we hypothesized that the substitution of cysteine (amino acid number 105, without signal peptide) by valine, as it occurs in the mutation C105Vfs114X, destroys one of the essential intramolecular disulfide bridges that structurally fixes the so called ”seat-belt” region involved in the receptor/ligand interplay [28]. The potentially affected disulfide bridge (Cys19-Cys105) is essential for the formation of the “seat-belt” conformation, which plays a role in the heterodimerization of CGA- and the TSHB-subunit [26]. Consequently, the pathogenic *TSHB* mutation may modify the TSHB structure, and possibly the heterodimeric hormone complex, which potentially leads to inactivation of the hormone or to modified signaling in interplay with the TSHR. The TSHR is able to activate all four G protein families [29,30,31,32,33]. For the Gq activation, it is known that higher concentrations of TSH are needed [29,30]. Although the mutation C105Vfs114X was functionally characterized through cAMP signaling induction at the TSHR, the mutation’s effect on the activation of other TSHR-specific signaling pathways [11] has not been investigated. Therefore, the aim of this study was to re-examine this mutation in a more comprehensive manner by testing the patient mutation and further mutants in a variety of pathways (Gs, Gq and MAPK). Through generating different mutants, we wanted to determine whether the shortened Ctt or the modified amino acid sequence could be responsible for structural modifications of the protein and eventually be related to the severe phenotype.

## 2. Results

### 2.1. Quantification of TSH-WT and Mutants

A prerequisite for testing the signaling properties of TSHB-WT and its mutants is their quantification. This is difficult, as TSH mutants are not recognized by most commercially available kits. Therefore, we took advantage of a recently developed new system to quantify these mutants [34]. 

**Figure 2:**
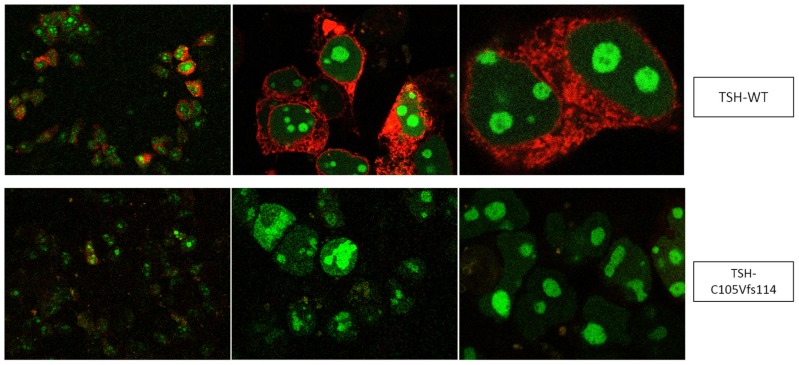

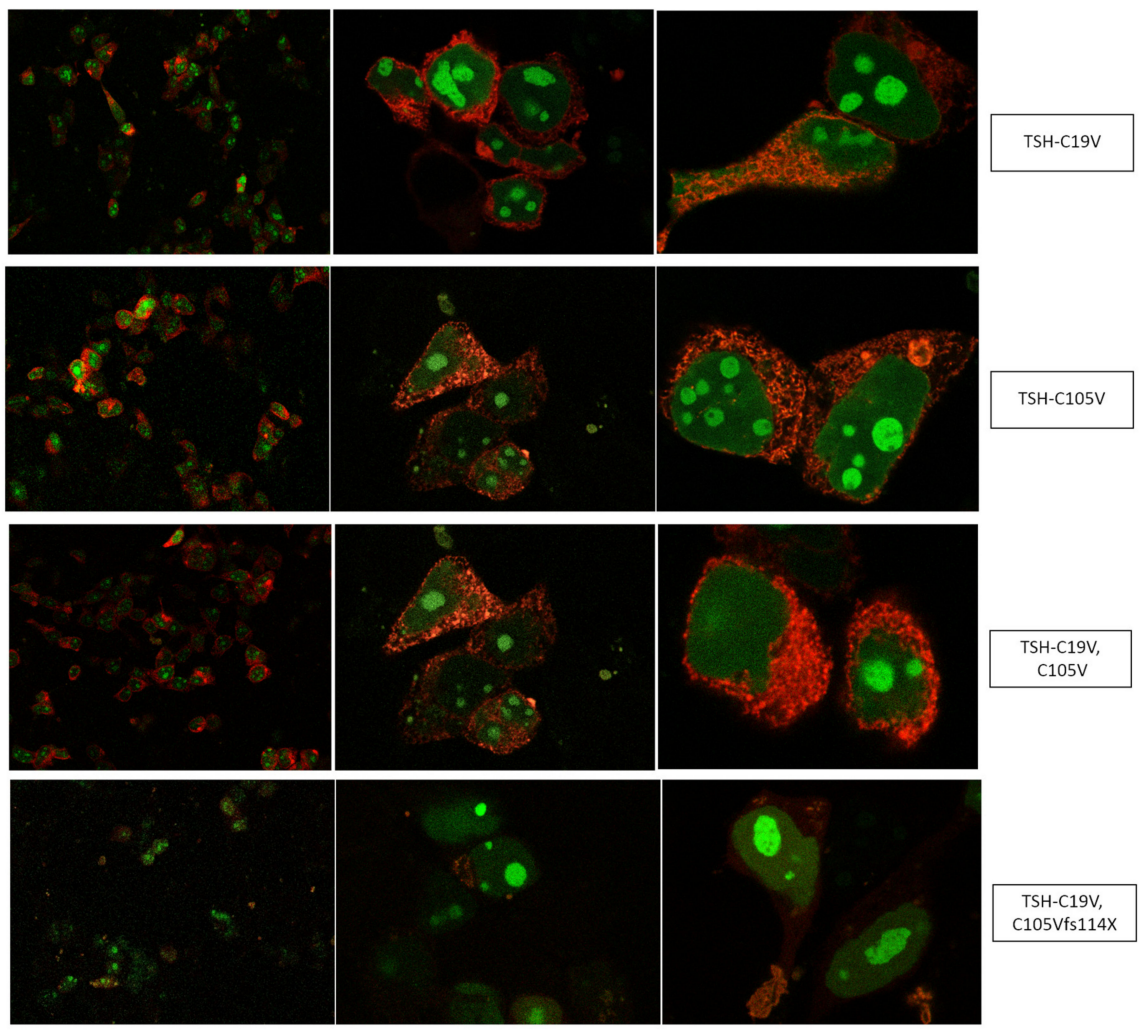
Confocal microscope images showing TSH expression at 20×, 40× and 63× magnification. HEK293 cells were transfected with the α- and β-subunit (latter cloned into the nAmCyan-P2A-mCherry vector) of WT-TSH and mutant TSH. The transfection efficiency and subsequent expression is shown in the green fluorescence (AmCyan). Since the red fluorescence (mCherry) is faded in the frameshift mutations, the comparison is based on the green fluorescent (AmCyan).

This system contains a vector, that encodes for two fluorescent proteins (AmCyan and mCherry). In addition, the plasmid contains a restriction site where a gene of interest (e.g., *TSHB*) can be inserted. AmCyan contains a nuclear localization signal (NLS) which moves into the nucleus (AmCyan) once the vector is expressed, while mCherry remains in the cytoplasm. In this way, AmCyan shows the levels of expression of the mRNA to protein, while mCherry monitors proper biosynthesis, transport and secretion [34]. For quantification, we used HEK293 cells, transfected with WT-TSH and mutants that were cloned into the vector nAmCyan-P2A-mCherry. The successful transfection and production of WT-TSH and the mutants were verified by visualization via a confocal microscope (Figure 2). 

**Figure A1:**
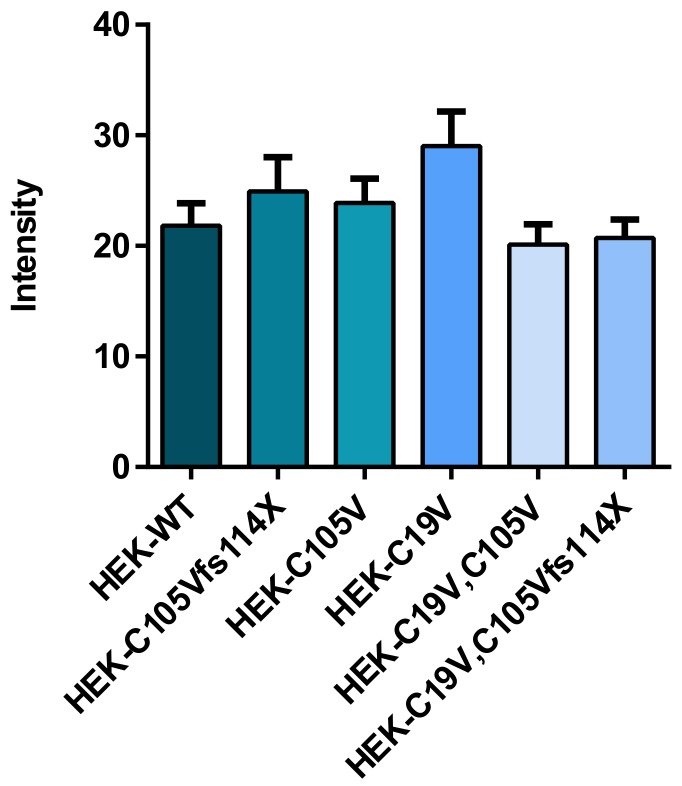
Comparison of the light intensity for quantification of WT-hTSH and mutants. For that HEK293 cells were transient transfected with WT-hTSH or the mutants (all cloned into the nAmCyan-P2A-mCherry vector). For comparison, the light intensity of AmCyan fluorescent protein was measured. Data are expressed as mean ± SEM of four independent experiments with 10 measurements each (*n* = 40). For statistical analysis, one-way-ANOVA with the Kruskal-Wallis test was used to test the mutants against the WT.

We additionally compared the transfection efficiency by measuring the light intensities of the nucleus. There, we also found no significant differences in the expression of the WT and the designed TSHB-mutants (Figure A1). Therefore, the amount of the WT-TSH was determined by using a commercial human TSH-ELISA (TSH (Human) ELISA Kit, Abnova, Taipei, Taiwan). For WT-TSH, the ELISA measured a concentration of about 20 mU/mL. Since the measured light intensities of the expressed AmCyan fluorescent protein of the mutants did not differ from the WT, the different mutants were estimated to have approximately the same concentration of 20 mU/mL.

**Figure A2:**
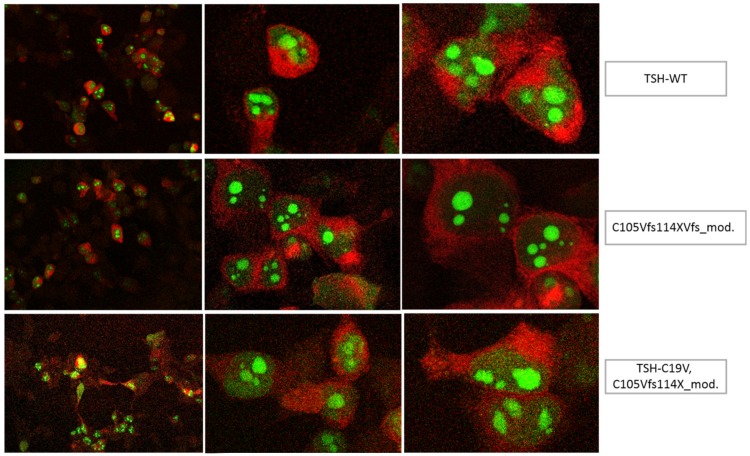
Confocal microscope images showing TSH expression at 20×, 40× and 63× magnification. HEK293 cells were transfected with the α- and β-subunit (the latter cloned into the nAmCyan-P2A-mCherry vector) of WT-TSH and modified *TSHB* mutants. The transfection efficiency and subsequent expression is shown in the green fluorescence (AmCyan). When comparing the WT-TSH and the two modified frameshift mutations no difference regarding the expression was seen.

Furthermore, we constructed two additional frameshift mutations (C105Vfs114X_mod. and C19V,C105Vfs114X_mod.) in order to rule out that the original frameshift mutations (C105Vfs114X and C19V,C105Vfs114X) lead to a mutated TSH, which might be degraded, aggregated or sequestered intracellularly and as a consequence is never secreted into the media. For that, we omitted the stop codon at position 114, which led to a fused TSHB in a similar way to mCherry as the WT. These modified frameshift mutations then showed an expression pattern of AmCyan and mCherry comparable to WT TSH (see Figure A2).

### 2.2. Functional Characterization

**Figure 3:**
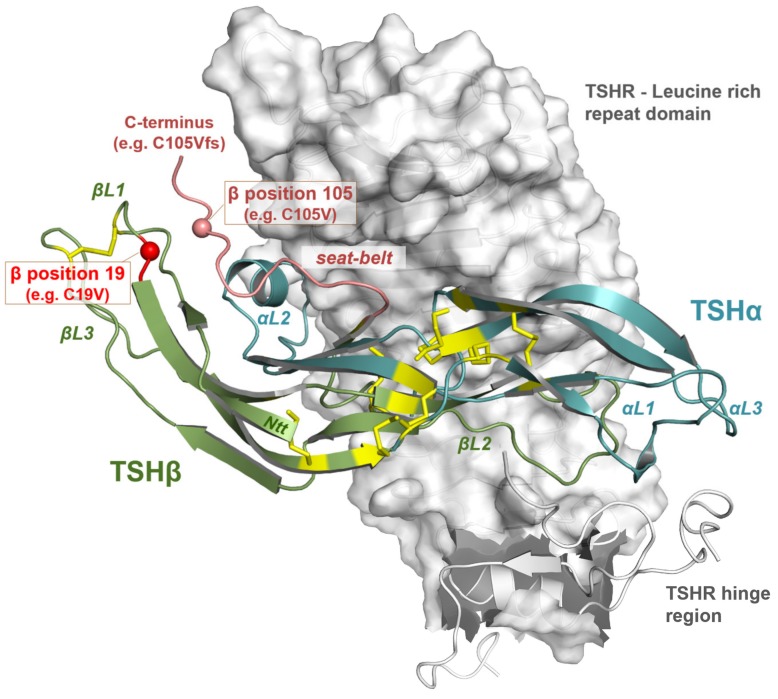
Homology model of bound TSH at extracellular TSHR components. This TSHR (partial, translucent surface representation of the LRRD)–TSH complex model was designed, as previously described [36], based on the solved X-ray structure of the follitropin receptor (FSHR) ectodomain with bound FSH (follicle-stimulating hormone) [37]. The orientation of TSH (WT) at the receptor is likely similar as observed for the homologous FSH at the FSHR. While we cannot predict how the TSH mutants in this study bind to the ectodomain, this model highlights respective features involved in the ligand binding and where the (pathogenic) modifications are spatially located in the hormone structure and in the formed putative complex. Details of the heterodimeric hormone structure are labeled, e.g., the loop regions of each subunit (alpha and beta) or the “seat-belt” region which are most likely justified by the WT disulfide-bridge between cysteins C19 and C105. The constructs tested in this current study are modified at these positions (Figure 3) and at the Ctt of the beta-subunit as observed in the patient mutant. Therefore, it must be assumed that the receptor/hormone complex can be different in detail comparing WT and modified TSH. TSH disulfide-bridges are represented as yellow sticks. For structure presentation and image production the PyMOL software was used (Molecular Graphics System, version 1.5; Schrödinger, LLC).

Our purpose was to more deeply understand the modifications at TSH caused by the patient mutation C105Vfs114X and the impact on signaling at the TSHR. This mutation leads to the loss of a Ctt cysteine that usually interacts with a N-terminally (Ntt) located cysteine (C19, numbering without signal peptide, Figure 1). This disulfide-bridge putatively stabilizes a structural unit called “seat-belt” (Figure 3), which is involved in the stabilization of a heterodimeric TSH structure [26]. Moreover, the frameshift at the Ctt leads to a modified sequence, which is also shorter compared to WT-TSH (Figure 1).

Of note, a shorter Ctt or a modified cysteine-bridge pattern can also be observed in thyrostimulin, the ancestral glycoprotein-hormone that stimulates TSHR highly efficiently (reviewed in [26,35]. 

**Figure 4:**
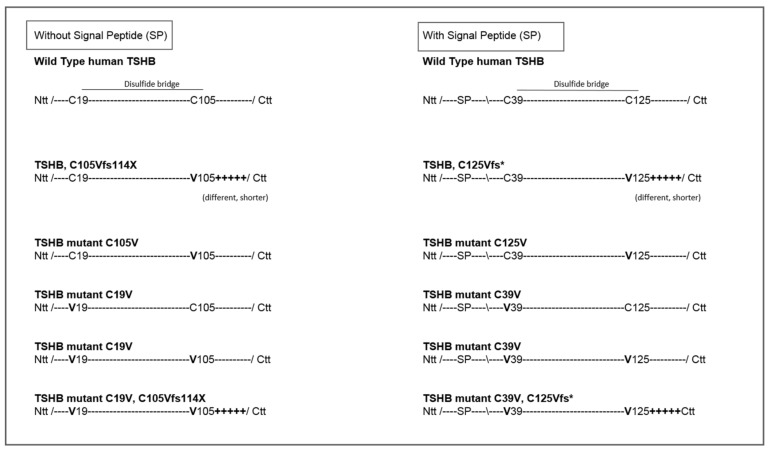
Different TSHB constructs used in this current study. This figure shows specific features of TSHB WT and tested mutations. Left: In this study we used the numbering system counted without the signal peptide. Right: WT and mutants are shown with SP, which leads to a different numbering of amino acids (20 amino acids more), while the constructs are finally identical compared to the left side. Ntt—N-terminus; Ctt—C-terminus, SP—signal peptide.

To estimate the exact functional consequence of each of these modifications at TSHB we designed and expressed several TSH mutants as presented in Figure 4. In principle, we assumed that the Ntt cysteine (C19 (or C39, numbering with signal peptide)) that usually interacts with the Ctt cysteine (C105 (or C125)) is free in the patient mutation and may interact with a different cysteine in the subunit and by this leads to a misfolded hormone subunit. In that case, a simultaneously mutated C19V and C105V should (even partially) reconstitute the TSH WT function (construct C19V/C105V). Moreover, at this construct we also modified the Ctt amino acids (C19V/C105Vfs) towards the patient mutation (Figure 4). These double mutations were accompanied by the respective single substitutions (constructs C19V, C105V) to evaluate their signal induction properties at the TSHR.

#### 2.2.1. Gs Mediated Signaling in HEK293 Cells

**Figure 5:**
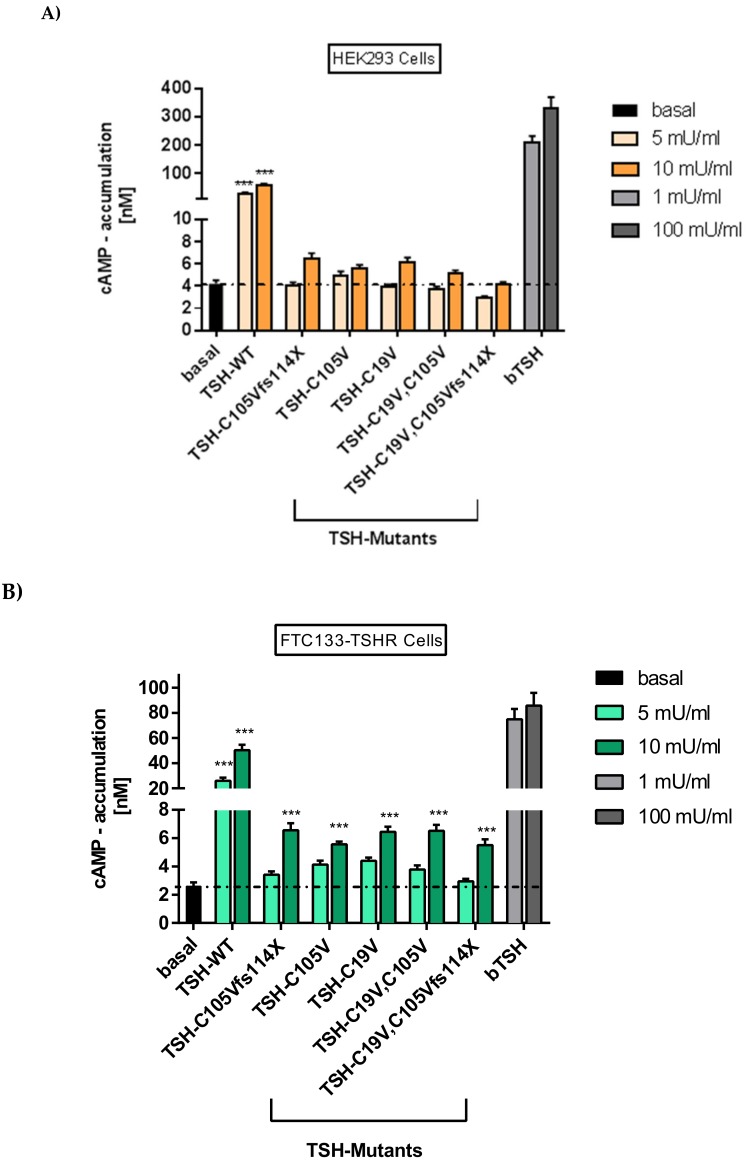
WT-TSH and TSH-variant signaling at TSHR in HEK293 cells and FTC133-TSHR cells. (**A**) Functional characterization of Gs/adenylyl cyclase signaling of WT-TSH and mutants at TSHR in HEK293 cells with different ligand concentrations. The basal activity of TSHR is shown as black bar/black line. (**B**) Functional characterization of Gs/adenylyl cyclase signaling in FTC133 cells. The basal activity is shown in black/black line. (A,B) bTSH functions as positive control. Data are expressed as mean ± SEM of four independent experiments performed in triplicate. For statistical analysis, one-way ANOVA with the Kruskal-Wallis test was used to test basal activity of the TSHR against the WT and TSH mutants. *** *p* < 0.001.

HEK293 cells transiently over-expressing TSHR were stimulated with both WT-TSH and TSH-mutants, and activation of Gs/adenylyl cyclase was measured. The activation of TSHR by bovine WT-TSH (bTSH) at a concentration of 1 mU/mL and human WT-TSH (hTSH) at a concentration of 10 mU/mLwas comparable. This is congruent with the fact that hTSH activates the TSHR with a 10-fold lower efficiency [38,39,40]. For the patient mutation and other TSH mutants, low but not significant increases in Gs signaling were noted by stimulation with 10 mU/mL hormone concentration. Neither the patient mutation, nor the further designed TSH mutants (Figure 4) led to a signal reduction below the basal signaling level, which excludes an inverse agonistic effect of the tested mutants (Figure 5A).

#### 2.2.2. Gs Signaling Pathway in FTC133-Cells

Signaling of WT-TSH and mutants was tested in the thyroid follicular carcinoma cell line FTC-133 (lacking endogenous TSHR expression) that were stably transfected with TSHR [41]. Gs-mediated signaling of the patient mutation and other mutants in these cells is higher compared to HEK293 cells, but lower compared to the WT-TSH and bTSH (Figure 5B). It was also observed that in FTC133 cells the patient mutation and none of the designed mutants showed a reduction of basal signaling of TSHR, which excludes an inverse agonistic effect.

**Figure A3:**
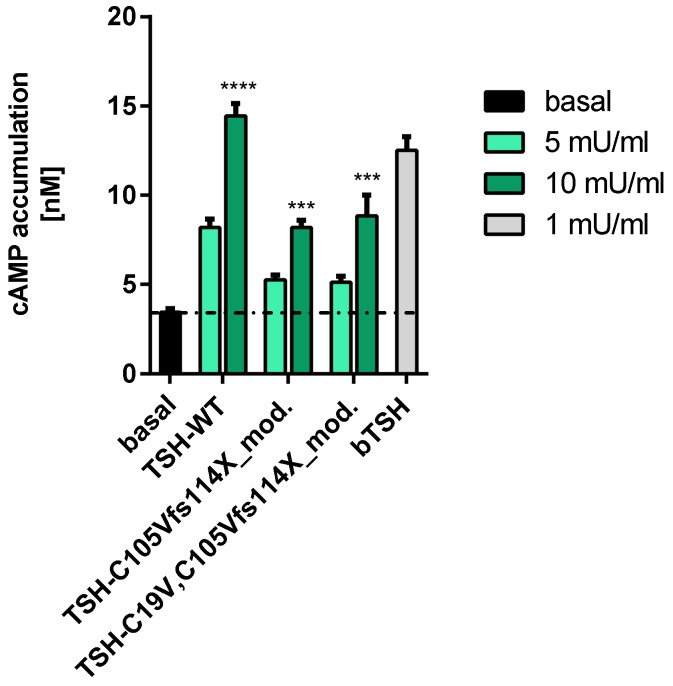
WT-TSH and modified mutant-TSH signaling at TSHR in FTC133-TSHR cells. Functional characterization of Gs/adenylyl cyclase signaling of WT-TSH and mutants at TSHR in FTC133 cells with different ligand concentrations. The basal activity of TSHR is shown as black bar/black line. bTSH (1 mU) functions as positive control. Data are expressed as mean ± SEM of four independent experiments performed in triplicate. For statistical analysis, one-way ANOVA with the Kruskal-Wallis test was used to test basal activity of the TSHR against the WT and TSH mutants. *p* *** < 0.001; **** *p* < 0.0001.

In an additional step, we performed a functional characterization of Gs/adenylyl cyclase signaling of the modified frameshift mutant TSH (fused to mCherry, see results 2.1) and WT-TSH at TSHR. For that, FTC133 cells were used since they represent the more physiological setting compared to HEK293 cells. As the positive control, we used 1 mU/mL bTSH. The results were similar to the previous conducted assays with the original frameshift mutations in FTC133 cells (Figure 5A,B). Again, the activation of the TSHR of 1 mU/mL (bTSH) and 10 mU/mL hTSH was comparable. The WT-TSH as well as the mutated TSH (at higher concentrations of 10 mU/mL) led to a significant increase of the Gs-mediated signaling, which was compatible with the previous results of Gs/adenylyl cyclase signaling in FTC133 cells (Figure 5A,B). Neither the WT-TSH nor the modified TSH showed a reduction of basal signaling at TSHR excluding an inverse agonistic effect of the modified TSH (Figure A3). 

#### 2.2.3. MAPK Signaling Pathway in HEK293 Cells

**Figure 6:**
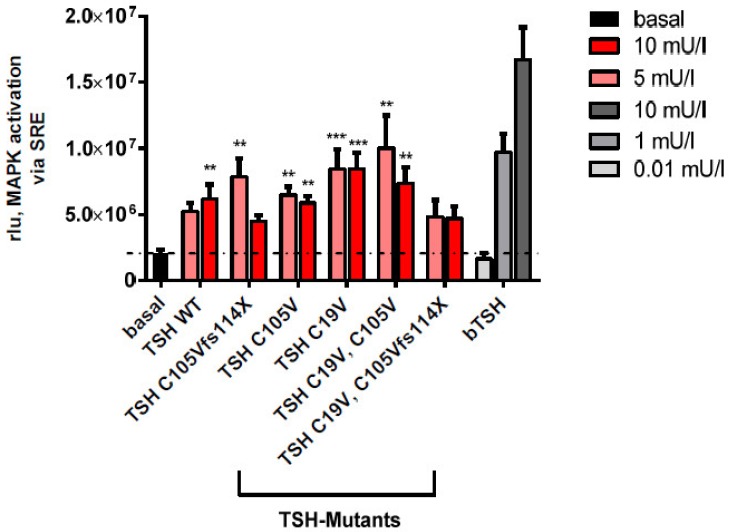
Determination of MAPK activation of WT-TSH and TSH mutants in HEK293 cells. Functional characterization (MAPK pathway) via a reporter gene assay with SRE. bTSH was used as positive control. Data are expressed as mean  ±  SEM of four independent experiments performed in triplicate. For statistical analysis, one-way ANOVA with the Kruskal-Wallis test tested basal signaling against all TSHB mutants. Statistical significance is indicated as ** for *p* < 0.01 and *** for *p* < 0.001.

To test the possibility that the patient’s phenotype (C105Vfs114X) is related to modifications in other pathways, MAPK activation was tested. This assay was performed in HEK293 cells co-transfected with both, TSHR and a reporter for MAPK activation (serum-response element; SRE) (Figure 6). In comparison to bTSH, 10 mU/mL hTSH demonstrated a reduced efficiency at TSHR. However, there was no significant difference in activation between WT-TSH and the hormone mutants at both tested concentrations (5 and 10 mU/mL). 

#### 2.2.4. Gq Mediated Signaling Pathway in HEK293 Cells 

**Figure 7:**
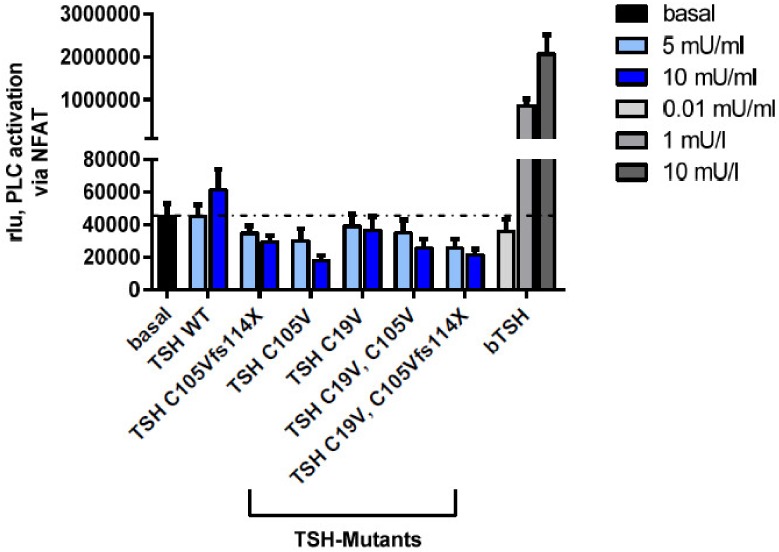
Determination of Gq/G11 Phospholipase C-β activation of WT-TSH and TSH mutants in HEK293 cells. Functional characterization (Gq pathway) via a reporter-gene assay with NFAT of the WT-TSH and tested mutants. Data are expressed as mean ± SEM of four independent experiments performed in triplicate. For statistical analysis, one-way ANOVA with the Kruskal-Wallis test for basal activity of the TSHR against the WT, all mutants at all concentrations.

It is known that the second important pathway of TSHR is the activation of Gq/11/Phospholipase C-β; however, higher concentrations of TSH are needed to activate this pathway [30,31]. For determination of the Gq signaling pathway, a reporter gene assay (nuclear factor of activated T-cells; NFAT) was used [42]. In contrast to bTSH, hTSH showed only a minor activation of the Gq signaling pathway (Figure 7), most likely due to TSH concentrations, which are too low to activate PLC signaling. This was true for all tested mutants. We speculate that substantially higher amounts of TSH protein would be necessary for stimulating this pathway. With the current methods it was unfortunately not possible to generate a sufficient amount of protein. 

## 3. Discussion

Patients with the mutation C105Vfs114X in the *TSHB*-gene show severe clinical signs of congenital hypothyroidism in the neonatal period and early infancy and psychomotor as well as neurological retardation if not detected and substituted with L-thyroxine early in life [13,15,16,19,21,25]. The mutation has been known for over 20 years, but the underlying molecular mechanisms have not yet been fully elucidated [26]. In this study, we tested the functional effects of this mutation as well as several other directed mutations to study the potential underlying molecular mechanims. 

### 3.1. Altered AmCyan-P2A-mCherry Expression Pattern of the Frameshift Mutations

We assumed that the faded red fluorescence (Figure 2) was due to the premature stop of the frameshift mutations (C19V,C105Vfs114X and C105Vfs114X), which results in a lacking fusion of TSH to mCherry. Since we were not able to rule out that the mutated TSH may be degraded, aggregated or sequestered intracellularly, and as a consequence is never secreted into the media, we constructed two additional mutations and modified them by omitting the stop codon resulting in a fusion to mCherry. The modified TSH-mutants then showed the same expression pattern as the WT and other tested mutants without the frameshift (Appendix B). Therefore, it was presumed that the mutated TSH is produced and secreted into the media. 

### 3.2. Signaling Capacities of the Mutation C105Vfs114X

The functional characterization of the modified mutations confirmed this assumption. Gs/adenylyl cyclase signaling of the modified TSH-frameshift mutations showed very similar results to the original frameshift mutations (C105Vfs114X and C19V, C105Vfs114X) (compare Figure 5 and Appendix C). We therefore presume that the frameshift mutations and especially the patient mutation C105Vfs114X lead to reduced Gs/adenylyl cyclase signaling compared to the WT. 

It is known that the TSHR is a promiscuous G protein-coupled receptor (GPCR) and initiates several distinct signaling pathways by binding of TSH [29,30]. We here found that activation of the Gs/adenylyl cyclase pathway through TSHR is diminished for the patient mutation as well as for all *TSHB* mutants designed to unravel the underlying molecular mechanism of the patients mutation (Figure 5). Of note, neither the patient mutation nor any of the TSH mutants reduce the basal Gs signaling capability of TSHR, ruling out an inverse agonistic effect (Figure 5).

Unfortunately, with the applied method, the TSH concentration of WT-TSH achieved was too low to induce a robust activation of Gq/11 phospholipase C signaling (Figure 7). Therefore, at this point we could only speculate that the loss of Gs and potentially Gq signaling together account for the severe phenotype of the patients with the specific *TSHB*-gene mutation C105Vfs114X. 

Interestingly, MAPK signaling of designed mutants (Figure 4) was unchanged, as compared to WT-TSH (Figure 6). Since the activation of MAPK is downstream of a variety of signaling pathways, we could not discriminate whether one or several pathways are unaffected with the applied method. However, since Gs signaling of WT-TSH is higher compared to investigated TSH mutants (Figure 5), one may speculate that Gi activation may be related to the observed MAPK signaling. Unfortunately, the role of Gi signaling induced by TSH at TSHR remains completeley elusive. However, the unmodified MAPK signaling might be seen as a hint that patient TSH mutation still binds at TSHR and induces signaling (Figure 6). This would be an argument against the hypotheses that the patient mutation primarily avoids heterodimer formation and non-binding at the TSHR. A single subunit of TSH, either alpha or beta, would likely not induce MAPK signaling as WT-TSH. In agreement, Chen et al. showed in directed deletion studies at the hormone CG (human chorionic gonadotropin), that the corresponding disulfide bridge in CG compared to the TSH modified in the patient hormone is not essential for heterodimer formation, receptor binding and subsequent intracellular signaling [43]. We conclude that further studies on *TSHB* mutants would be necessary to compare signaling properties of those mutants to finally dissect the underlying cause of variations in the patients’ phenotypes.

### 3.3. Impact of Potential TSH Structure Modification by Amino Acid Variations

In this study, we performed extended experiments to unravel the role of the mutated cysteine at position 105 to valine or regarding the modified Ctt amino acid sequence in the *TSHB*-mutation C105Vfs114X. Through the substitution of cysteine by valine, an essential disulfide bridge is disrupted and the remaining cysteine should cause a “false” disulfide-bridge with another cysteine in the sequence. Such scenario would lead to a 3D-TSH structure that is different to the WT. Of note, this assumption was supported by the fact that thyrostimulin endogenously lacks not only the Ctt cysteine, but also the Ntt cysteine at the corresponding position [26].

Our experiments showed that the designed mutation C19V as well as the double mutation C19V/C105V that lacks both cysteine residues has similar effects on the Gs mediated signaling at the TSHR as the patient mutation C105Vfs114X (Figure 5, Figure 6 and Figure 7). We therefore conclude that a free cysteine at the Ntt is not contributary to functional, and possibly structural, changes. Moreover, the triple mutant C19V/C105Vfs114X with a diverse Ctt sequence shows no difference in signaling compared to the single or double mutation (Figure 5 and Figure 6). Interestingly, Mishra et al. investigated the heterodimerization of the hCG beta subunit with the alpha-subunit. They showed that only three of the six investigated cysteine bridges play a crucial role for the heterodimerization and subsequently for the correct hormone function [44]. A study on CG and LH (lutropin, luteinizing hormone) supports the hypothesis of biased signaling induced by hormone variants at one receptor [45]. They demonstrated an endogenous biased agonism by comparing the signaling of LH and hCG at the LHCGR. The two hormone variants showed differences in terms of potency, efficiency and kinetics at their common receptor. A significant structural difference of these two hormones is the length and composition of the Ctt. Therefore, it can be speculated that differences of the Ctt (including the disulfide-bridge) of *TSHB* mutants compared to WT *TSHB* might also contribute to biased signaling at the TSHR as observed here.

Moreover, the known thyrostimulin-induced cAMP signaling without such corresponding cysteine-bridge indicates that the general TSH orientation at the TSHR in the bound state may not be significantly shifted without this disulfide bridge [46]. However, the detailed interactions between receptor and hormone responsible for final Gs signal induction by hTSH are hindered by the loss of this specific disulfide-bridge. This assumption is supported by the single mutations C19V (or C39V) or C105V (C125V) that are similarly diminished in the capacity for cAMP signaling induction at the TSHR as all other mutants, but they are still able to induce MAPK signaling via the TSHR.

### 3.4. Comparison with Other Pathogenic Pituitary Hormone Mutations

Although rare, several glycoprotein hormone mutations at LH, FSH and CG were already identified. A few of them are localized proximal to the Ctt of the beta-subunit or involving a cysteine bridge and have shown to have an impact on the binding affinity and potency at the receptor as well as on the bioactivity of the hormone [47]. A case study by Misgar et al. revealed a Ctt *FSHB* mutation (c.343C>T:pArg115Stop) that ends in a premature stop (comparable to our patient mutation) [48]. This mutation results in an isolated FSH-deficiency with absent breast development and primary amenorrhea in females and azoospermia with normal testosterone levels in males. Of particular interest were two mutations in *FSHB* (Cys51Gly and Cys82Arg) that are involved in forming the cysteine-knot-like structure of the protein (again comparable to our patient mutation) and also lead to FSH-deficiency with female sexual infantilism and infertility in female and azoopermia but normal masculinization in male [47]. On the contrary, a study of three naturally occurring heterozygous *CGB* mutations (CGB5 p.Val56Leu; CGB8 pPro73Arg; CGB8 p.Arg8Trp) demonstrated that these mutations only lead to mild structural and functional impairments, hypothesizing that more severe impairments would lead to a complete pregnancy failure [49]. 

Taken together, these comparisons show that especially homozygous mutations localized in the Ctt region or involving disulfide bridges can lead to an impaired heterodimerization, a reduced binding affinity or altered intracellularly signaling, resulting in a severe phenotype. Mutations that do not involve cysteine bridges or are not localized proximal to the Ctt seem to show milder structural effects resulting in a less severe phenotype [49,50].

### 3.5. Clinical Features of the Mutation C105Vfs114X in Comparison with Other TSHB Mutations

As already mentioned, the mutation C105Vfs114X leads to extraordinary early and severe signs of congenital hypothyroidism compared to patients with a complete absence of TSH [4,5,6,7]. This was surprising because the TSHR shows constitutive basal signaling activity, which should be responsible for a minimal level of Gs signaling activity and subsequent thyroid hormone production. In turn, it might be assumed that this specific *TSHB* mutation C105Vfs114X should lead to an inverse agonist effect at the TSHR as observed for specific antibodies as well [51,52]. This theoretical hypothesis has never been tested before, but can be excluded based on our data presented here (Figure 5). In addition, this current study shows that even the modified TSH leads to a low level of TSHR activation. 

However, the full explanation for the severe clinical effects of the C105Vfs114X mutation, especially also in contrast to other *TSHB* mutations phenotypes, needs further elucidation. For example, three children with the mutation E12X in the *TSHB* were diagnosed at the age of 3.5, 5 and 9 months and showed a normal development [53]. The same applies to a child with the mutation C85R that was diagnosed with congenital hypothyroidism and substituted with L-thyroxine at the age of 6 months, but did not present with any long-term deficiencies [50]. In contrast, patients that were diagnosed with the mutation C105Vfs114X at the age of 2 months and substituted with thyroid hormones immediately, showed at least a mild retardation at a follow-up examination [15,20]. Children that were diagnosed later suffered from even more severe mental retardation up to an IQ of 66 [19,25]. 

Based on the varied observations and on our data, we speculate that different *TSHB* mutations may lead to different signaling properties on the TSHR and consequently to diverse phenotypes. Of note, to our knowledge, none of the other *TSHB* mutations identified so far has been systematically characterized in vitro for signaling pathway modifications. The available case descriptions, which are primarily based on clinical observations, do not allow a systematic comparison or the exact assignment of TSH/TSHR induced signaling pathways and distinctive physiological impacts. Therefore, further studies are necessary to compare naturally occurring *TSHB* mutations and related phenotypes. 

## 4. Materials and Methods 

### 4.1. Design of TSH-Mutants and Cloning Strategy

TSH mutants including the patient mutation were designed according to the scheme presented in Figure 4 and cloned into the AmCyan-P2A-mCherry vector. This vector encodes for two fluorescent proteins (AmCyan and mCherry) and functions, as already mentioned in the result part. For generating the various mutants, a synthetic gene fragment (Integrated DNA Technologies, Coralville, IA, USA) containing the *TSHB*-WT was cloned into the nAmCyan-P2A-mCherry vector [34]. *THSB* mutants were then generated from the WT through either the REPLACR mutagenesis method [54] or for the double mutants by Gibson Assembly cloning kit (New England Biolabs, Ipswich, MA, USA) using the following primers (Table 1). All clones were verified by Sanger-Sequencing.

### 4.2. Cell Culture and Transfection

All cells were cultivated in a humidified 5% CO2 incubator at 37 °C using various media and supplements. HEK293 (American Type Culture Collection, Manassas, VA, USA) cells were used for protein production and functional characterization. FTC133-cells [55] were only used for functional characterization in order to create an environment closer to human physiology. For protein expression, HEK293 cells were cultivated in 1:1 Dulbecco’s modified medium (DMEM/Ham’s F12 medium (Biochrom GmbH, Berlin, Germany) supplemented with 5% charcoal treated serum. HEK293 cells for functional characterization were grown in Dulbecco’s MEM with stable glutamate (Biochrom GmbH, Berlin, Germany) and supplemented with 5% fetal bovine serum (FBS) (Biochrom GmbH, Berlin, Germany) and non-essential amino acids (NEA) (Biochrom GmbH, Berlin, Germany). FTC133 cells with stably expressed TSHR were cultivated in Iscove Basal medium (Biochrom GmbH, Berlin, Germany) supplemented with L-glutamine, 10% FBS, 1% penicillin/streptomycin (all Biochrom GmbH, Berlin, Germany). For protein expression of WT-TSH and the mutants, HEK293 cells (4.5 × 10^6^) were seeded in 175 cm^3^ flasks (Sarstedt, Nuembrecht, Germany). For proving the successful expression of WT-TSH and mutated TSH, 1.5 × 10^5^ cells/well were additionally seeded in 6-well-plates (containing cover glasses) and Fluorodishes (Thermo Fisher Scientific, Waltham, MA, USA). Twenty-four hours later, the plasmids encoding the common α-subunit (cloned into the pcDNA3 vector) and the specific β-subunit (WT-TSH and TSH mutants cloned into the nAmCyan-P2A-mCherry vector) were transfected with Metafectene (Biontex, Munich, Germany) into the cells with a DNA 3:1 ratio (66 μg (2.4 μg) α-subunit: 22 μg (0.8 μg) β-subunit per flask (well)). For functional characterization, HEK293 cells were transfected with TSHR (cloned into pcDps vector). Cells were cultivated as described above and 1 × 10^4^ HEK293 seeded in 96-well plates. Twenty-four hours later, the cells were transfected with TSHR plasmid DNA (45 ng/well) and Metafectene (Biontex, Munich, Germany) (0.45 µL/well), cultivated in supplement-free DMEM medium (Biochrom GmbH, Berlin, Germany). Forty-eight hours later, the cells were stimulated. Since the FTC133 cells were stably expressing the TSHR, they were seeded in 96-well plates at a cell density of 1 × 10^4^ and stimulated after 24 h. For the reporter-gene assays, HEK293 cells were seeded at a density of 1 × 10^4^ per well and after 24 h co-transfected with equal amounts (96-well plate; 45 ng/well) of plasmid DNA (TSHR) and the reporter gene construct SRE (cloned into pcDps vector) for MAPK and NFAT (cloned in pcDps vector) for Gq signaling. Forty-eight hours later, the transfected cells were stimulated with WT-TSH or the mutations.

### 4.3. Protein Expression and Quantification

HEK293 cells were seeded and transfected after 24 h (according to the method described above). Twenty-four hours after that, the successful production of TSH was proved. For that, the cover glasses were fixed and images were rendered using a TCS SPE Confocal Microscope (Leica, Wetzlar, Germany) and the software Application Suite V4.9 (Leica, Wetzlar, Germany) for image analysis. Using Fluorodishes, the light intensities of the AmCyan fluorescence protein (higer intensities indicated higher transfection efficiency) in WT-TSH and mutant TSH were measured. This was conducted using the light microscopy Axiovert 10 (Zeiss, Oberkochen, Germany) and the software Live Aquisitions V2.6.0 (FEI Munich GmbH, Munich, Germany). These two methods showed that there is no difference in the produced amount of WT-TSH and mutated TSH. Therefore, 48 hours after the transfection, the supernatants of the 175 cm^3^ flasks that contained the WT-TSH and mutant TSH, were collected and purified. The purification was conducted by an ultrafiltration of the supernatants using the Amicon Ultracel Filter System (Merck, Darmstadt, Germany) with a molecular weight cut-off of 3 kDa, meaning that all molecules with a molecular weight over 3 kDa (including TSH with a molecular weight of ~28.5 kDa) were not filtered. The amount of the concentrated WT-TSH was then measured with the commercial TSH (Human)-ELISA Kit (Abnova, Taipei, Taiwan). Preliminary testings showed that this was not possible for TSH mutants, most likely due to the fact that the used antibody could not bind to the modified protein. Therefore, we used the quantification method described above and compared the produced amount by confocal microscopy and light microscopy. The results showed no difference, and therefore the amount of WT-TSH determined by the ELISA was equated with the amount of mutated TSH and used for the functional characterization. 

### 4.4. Functional Characterization

Two different assay systems were used for functional characterization. To test the Gs signaling pathway, cAMP accumulation was measured in HEK293 expressing TSHR. To examine Gs signaling also in a more physiological set–up, thyroid carcinoma cells (FTC133 cells), stably transfected with TSHR, were used. HEK293 cells were transiently transfected with the TSHR. As positive control, bTSH at concentrations of 100 and 1 mU/mL was used. For stimulation, TSH preparations and bTSH were diluted in stimulation buffer (138 mM NaCl, 6 mM KCl, 1 mM MgCl_2_, 5.5. mM glucose, 20 mM HEPES, 1 mM CaCl2 and 1 mM 3-isobutyl-1-methylxanthine (IBMX; Sigma Aldrich, St. Louis, MO, USA) and stimulation was performed for 45 min followed by cell lysis for 90 min at 4 °C. The accumulation of cAMP was determined using the AlphaScreen™ technology (Perkin Elmer, Life Science, Waltham, MA, USA) as previously reported [56].

To determine the MAPK and Gq signaling pathway, reporter-gene assays were used. Forty-eight hours after transfection, the cells were stimulated with WT-TSH and the mutant TSH, as well as with the positive control bTSH (Sigma Aldrich, St. Louis, MO, USA) (in concentrations of 100, 1 and 0.01 mU/mL) for 6 h at 37 °C. The cells were lysed for 15 min at room temperature using 50 µL Lysis Buffer (Promega, Madison, WI, USA) per well. The intracellular IP3 formation was then measured via the luciferase reporter-gene assay (Promega, Madison, WI, USA) as previously reported [42].

### 4.5. Software

The cAMP-assay, as well as the luciferase-assay, were measured by the Mithras LB 940 Multimode Plate Reader (Berthold Technologies, Bad Wildbad, Germany), using the software Mikrowin V.2000 (Labsis Laborsysteme GmbH, Neunkirchen-Seelscheid). The mathematical calculations of the raw data were conducted by Microsoft Excel. The statistical analysis, as well as the diagrams, were made with the software Graph Pad Prism V.6 (Graph Pad Software, San Diegeo, CA, USA). 

### 4.6. Statistical Analysis

For statistical analysis, we used the software Graph Pad Prism V.6 (Graph Pad Software, San Diego, CA, USA). Descriptive statistics were computed for all variables. These include means, standard deviations and standard errors. A nonparametric Kruskal-Wallis-Test was done to assess differences between the basal activity of the TSHR and the activation of the TSHR by WT-TSH or mutated TSH at different concentrations.

## 5. Conclusions

The in depth study of the naturally occurring human mutation C105Vfs114X in the *TSHB* gene, which unexpectedly results in an early and severe phenotype, might give major insights into the signaling capacities of the TSHR. Although much has been learned about TSHR structure and function in the last two decades, the specific molecular mechanisms of activation and inactivation are not fully understood. Thus, molecular lesion of *TSHB* resulting in a specific human phenotype can be a key element to elucidate mechanisms, which would otherwise remain unknown. The impact for translational research beyond the understanding of the basic biology might be the development of small molecules that very specifically can inhibit the TSHR signaling e.g., in the presence of TSHR antibodies in autoimmune thyroid disorders such as Grave’s disease. 

## Figures and Tables

**Table 1:** Table of the used primers for site-directed mutagenesis (bold letters indicate site of mutagenesis; mutation names including signal peptide).

	Mutation	Forward Primer	Reverse Primer
1	TSHB_C105Vfs114X	TSHB_C105Vfs114X_FP CCATCAAGACAAACTAC**GT**ACCAAACCTC	TSHB_C105Vfs114X_C_V_RP GTAGTTTGTCTTGATGGCTTCATGTATGC
2	TSHB_C105V	TSHB_C105V_FP CCATCAAGACAAACTAC**GTT**ACCAAACCTC	TSHB_C105V_C_V_RP GTAGTTTGTCTTGATGGCTTCATGTATGC
3	TSHB_C19V	TSHB_C19V_FP GGAGAGAGTGTGCTTAT**GTC**CTAACCATC	TSHB_C19V_RP GACATAAGCACACTCTCTCCTTTCGATG (CATCGAAAGGAGAGAGTGTGCTTAT**GTC**)
4	Lin_TSHB (Linearized vector for Gibson Assembly cloning)	Lin_TSHB_Gibs_FP ACCAAACCTCAGAAGTCTTATCTGG	Lin_TSHB_Gibs_RP ATAAGCACACTCTCTCCTTTCGATG (CATCGAAAGGAGAGAGTGTGCTTAT)
5	TSHB_C19V_ C105V (Insert)	TSHB_C19V_FP GGAGAGAGTGTGCTTAT**GTC**CTAACCATC	TSHB_C19V_ C105V_RP GACTTCTGAGGTTTGGTAACGTAGTTTGTC (GACAAACTAC**GTT**ACCAAACCTCAGAAGTC)
6	TSHB_ C19V_C105Vfs114X (Insert)	TSHB_C19V_FP GGAGAGAGTGTGCTTAT**GTC**CTAACCATC	TSHB_ C19V_C105Vfs114X_RP GACTTCTGAGGTTTGGTACGTAGTTTGTC (GACAAACTAC**GT**ACCAAACCTCAGAAGTC)
7	TSHB_C105Vfs114X_mod. (Insert)	TSHB_C105Vfs114X_mod.FP CCTCAGAAGTCTTATCTGGTA**T**GGATTTTCTGTCGTGAGCAAG	TSHB_C105Vfs114X_mod.RP CTTGCTCACGACAGAAAATCCAT**A**CCAGATAAGACTTCTGAGG
8	TSHB_C19V_C105Vfs114X_mod(Insert)	TSHB_C19V, C105Vfs114X_mod.FP CCTCAGAAGTCTTATCTGGTA**T**GGATTTTCTGTCGTGAGCAAG	TSHB_C19V, C105Vfs114X_mod.RP CTTGCTCACGACAGAAAATCCAT**A**CCAGATAAGACTTCTGAGG

## Abbreviations

ANOVA	Analysis of Variance
bTSH	bovine Thyroid Stimulating Hormone
cAMP	cyclic Adenosine Monophosphate
CCH	Central Congenital Hypothyroidism
CGA	α-Subunit of Glycoproteinhormones (including TSH)
CGB	β-subunit of the Chorionic Gonadotropine
Ctt	C-Terminus/C-terminal
ELISA	Enzyme-linked Immunoabsorbent Assay
FSH	Follicle-Stimulating Hormone
FSHB	β-subunit of Follicle-Stimulating-Hormone
FTC cells	Follicular Thyroid Carcinoma Cells
GPCR	G-Protein Coupled Receptor
HEK cells	Human Embryonic Kidney Cells
hCG	human Chorionic Gonadotropine
hTSH	human Thyroid Stimulating Hormone
LH	Luteinizing Hormone
LHB	β-subunit of Luteinizing Hormone
MAPK	Mitogen-Activated Protein Kinases
NFAT	Nuclear Factor of Activated T-Cells
Ntt	T-Terminus/N-terminal
SRE	Serum Response Element
TSH	Thyroid Stimulating Hormone
TSHB	β-subunit of TSH
TSHR	Thyroid Stimulating Hormone Receptor
WT	Wildtype
